# Self-Reported Asthma Is Associated with Reduced Sperm Count—A Cross-Sectional Study of More than 6000 Young Men from the General Population

**DOI:** 10.3390/life13020278

**Published:** 2023-01-19

**Authors:** Marc K. Pedersen, Elvira V. Bräuner, Ann H. Hansen, Laura S. Hansen, Tina K. Jensen, Niels Jørgensen, Lærke Priskorn

**Affiliations:** 1 Department of Growth and Reproduction, Copenhagen University Hospital—Rigshospitalet, 2100 Copenhagen, Denmark; 2 International Centre for Research and Research Training in Endocrine Disruption of Male Reproduction and Child Health (EDMaRC), Copenhagen University Hospital—Rigshospitalet, 2100 Copenhagen, Denmark; 3 Research Unit of Clinical Pharmacology, Pharmacy and Environmental Medicine, University of Southern Denmark, 5000 Odense, Denmark

**Keywords:** asthma, testicular function, semen quality, reproductive hormones

## Abstract

Asthma is driven by an inflammatory response that may impact testicular function. In this cross-sectional study, we investigated the association between self-reported asthma and testicular function (semen parameters, reproductive hormone levels), and determined whether potential further inflammation due to self-reported allergy modified this association. A total of 6177 men from the general population completed a questionnaire including information on doctor-diagnosed asthma or allergy, had a physical examination, delivered a semen sample, and had a blood sample drawn. Multiple linear regression analyses were performed. A total of 656 (10.6%) men reported having ever been diagnosed with asthma. Generally, self-reported asthma was consistently associated with a poorer testicular function; however, few estimates were statistically significant. Specifically, self-reported asthma was associated with statistically significant lower total sperm count [median: 133 vs. 145 million; adjusted β (95% CI): −0.18 (−0.33 to −0.04) million on cubic-root-transformed scale] and borderline statistically significant lower sperm concentration compared with no self-reported asthma. The association between asthma and total sperm count was of similar magnitude among men with and without allergy. In conclusion, men with self-reported asthma had poorer testicular function than men without asthma. However, the cross-sectional design of the study limits ascertainment of causality.

## 1. Introduction

Asthma is a prevalent disease of the lungs and airway, which can be either allergy- or non-allergy-induced [1,2]. Among men of fertile age, asthma is one of the most common chronic non-communicable diseases, presenting in up to 8% of men, and around one in four men report at least one allergy [3]. Asthma is driven by an inflammatory response which may adversely affect male reproductive organs, as illustrated in animal models [4,5]. Based on a well-established mouse model for asthma, asthmatic mice were found to have reduced testis weight, sperm count, and motility compared with control mice, and showed increased spermatocyte apoptosis, structural changes in spermatozoa, delay in sperm maturation, and inhibition of Sertoli cell function [6].

Over the past few decades, semen quality has been reported to be in decline [7], and a Danish study reported that only one in four men had an optimal sperm concentration above 40 million/mL and more than 9% normal forms [8]. Male semen quality is important for a couple’s fertility chances, and the probability of conception increases with increasing sperm concentration up to 40–50 million/mL [9,10]. Also, sperm motility and morphology are important, and high levels of one semen parameter cannot entirely compensate for low levels of the others [11]. Whether asthma contributes to male infertility via impairment of testicular function is unknown, but in women, an association between asthma and reduced fertility has been suggested [12]. A study among sub-fertile couples found that male users of asthma medication (sympathomimetic-users) had better sperm motility than non-sympathomimetic-users [13], but the role of asthma per se in this association was unclear. Due to the high prevalence of both asthma and poor semen quality, any association between the two has public health relevance and could have implications for the clinical management of infertility. Likewise, little is known about the potential effect of allergy on the association between asthma and testicular function. But because allergy induces inflammation and asthma exists as either allergic or non-allergic, an increased systemic inflammation could be hypothesized in men suffering from both conditions, potentially causing synergistic effects on the reproductive function [4].

By utilizing a population of young Danish men from the general population, we therefore investigated the association between self-reported asthma and testicular function and whether this was modified by self-reported allergy.

## 2. Material and Methods

Study design and data source: We performed a cross-sectional study based on the Danish Young Men’s Study (DYMS), which is a cohort of males that have well characterized testicular function. The DYMS was carried out to monitor semen quality of young men in Denmark and to study potential risk factors for impaired semen quality [8,14]. The men were invited to participate at the time of their compulsory physical examination performed prior to consideration for military service. This invitation was independent of being considered fit for military service, and the majority had no knowledge of their fertility status and are considered representative of the background population. The study was conducted at the Department of Growth and Reproduction, Rigshospitalet. All men delivered a semen sample, had a blood sample drawn, underwent a physical examination, and answered a comprehensive questionnaire.

**Figure 1:**
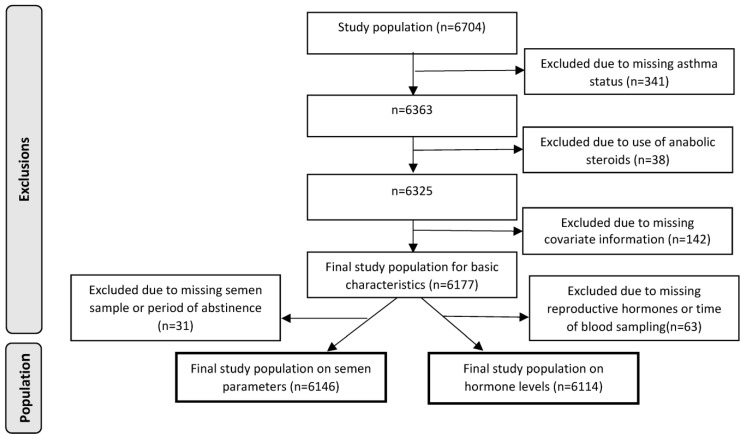
Flowchart of the study population.

Population: A total of 6704 men participated in DYMS between 2001 and 2020, but a total of 521 were excluded [missing information on asthma status (n = 341), use of anabolic steroids (n = 38), and missing covariate information (n = 142)] from the present study, resulting in a study population of 6177 men (Figure 1).

Questionnaire data, including self-reported asthma and allergies: The questionnaire included questions regarding health (disease and current regular use of medication), health behavior, and fetal exposure to maternal smoking. Use of medication was defined as daily use for at least a week within the last three months prior to study participation, and the men provided the product name and/or indication. For determination of ever-diagnosed asthma, in all years from 2001–2020, men were asked (in Danish): “*Have you ever been diagnosed with one or more of the following diseases by a medical doctor?*” followed by a list of specific diseases (yes/no). From 2001 to 2007, the question combined asthma/chronic bronchitis in a single item and did not include allergy, and in more recent years from 2008 to 2020, asthma, chronic bronchitis, and allergy were all included as separate items. For comparability between the two periods, the 26 males that answered yes to chronic bronchitis from 2008 to 2020 were categorized as self-reported asthmatic cases.

Physical examination: During the physical examination, testicular size was measured by a trained andrologist [Prader Orchidometer (volume) and ultrasonography, after which volume was calculated using the formula of an ellipsoid, ⅓π × L × W × H × ½] [15]. Weight and height were measured, and body mass index (BMI) was calculated using the formula, weight (kg)/[height (cm)]^2^. The andrologist had no knowledge of the participants’ semen quality, concentrations of reproductive hormones, or disease history at the time of examination.

Semen quality: Participants provided a semen sample by masturbation in a room located near the semen laboratory. They were instructed to abstain from ejaculation for at least 48 h prior to masturbation, and the exact duration of abstinence was recorded. The sample was analyzed for semen volume (assessed by weighing), sperm concentration (Bürker-Türk haemocytometer), percent of progressive motile spermatozoa (assessed on a clean glass slide), and percentage of morphologically normal spermatozoa (assessed according to strict criteria on Papanicolaou stained smears), in accordance with the 2010 WHO criteria and described previously [8,16]. Total sperm count was calculated using the formula, semen volume (mL) × sperm concentration (sperm count/mL).

Reproductive hormone levels: Blood samples were drawn from a cubital vein. Blood for analysis of reproductive hormones was centrifuged and the serum was stored at −20 °C until the time of analysis, as described elsewhere [17]. In brief, all hormones were analyzed in the same laboratory and in yearly batches. To assess serum levels of luteinizing hormone (LH), follicle stimulating hormone (FSH) and sex-hormone-binding globulin (SHBG) time-resolved immunoflouremetric assays (Delfia; Wallac, Turku, Finland) were used. For the analysis of total testosterone (T), time-resolved fluoroimmunoassay (Delfia; Wallac) was used. In the period from 2014 onwards, SHBG and T were measured by ELISA (Access 2; Beckman Coulter Ltd., High Wycombe, UK). Inhibin B was analyzed by a specific two-sided enzyme immunometric assay (Inhibin B Gen II; Beckman Coulter Ltd., High Wycombe, UK). Free T was calculated according to the equation suggested by Vermeulen based on measured serum levels of total T and SHBG and assuming a fixed albumin level [18].

Statistical analysis: First, descriptive statistics on anthropometric measures and lifestyle factors were calculated, stratified by self-reported asthma. Differences between the groups were assessed using the Mann–Whitney *U* test for continuous variables and Chi^2^ test for categorical variables. Semen parameters and reproductive hormones were similarly stratified according to asthma status for descriptive statistics.

We conducted linear regression to assess the association between self-reported asthma and semen parameters, as well as reproductive hormone levels. The outcomes—semen parameters and reproductive hormones—were included as continuous variables, transformed accordingly to pass the model assumption of linearity, normally distributed residuals, and homoscedasticity. Semen volume, sperm motility, and morphology were square-root-transformed, and sperm concentration and total sperm count were cubic-root-transformed. All hormones were transformed to their natural logarithm.

Analyses were conducted with and without adjusting for covariates selected *a priori*, based on available literature, and included potential confounders as well as precision variables that are only closely associated with the outcome measurement. All adjusted analyses included BMI (kg/m^2^, linear), current smoking (yes/no), and maternal cigarette smoking during pregnancy (yes/no). Regression analyses considering semen outcome parameters furthermore included ejaculation abstinence time (hours, linear), and analyses of motility additionally the duration between ejaculation and assessment of motility (minutes, linear), while regression analyses of reproductive hormone levels included time of blood sampling (hour and minute, linear). Respectively, 31 and 63 men were excluded from the regression analyses of semen parameters and reproductive hormone levels due to missing information on the outcome or related precision covariate (Figure 1). In total, 6146 men were included in the analyses of semen parameters, and 6114 men were included in the analyses of reproductive hormone levels (Figure 1).

To assess the robustness of estimates in our main models, we performed several sensitivity analyses. First, based on the 3487 men examined from 2008, and with detailed separate information on self-reported asthma and allergy, we tested the potential effect modification of allergy on the association between asthma and total sperm count by stratifying the adjusted analysis by allergy status (yes/no) and introducing an interaction term (asthma × allergy) into the non-stratified adjusted model to obtain the *p*-value for the interaction. Second, in the same population, we ascertained the potential contribution of chronic bronchitis to the estimates in our main analyses by repeating the analyses after excluding the 26 men with chronic bronchitis. Third, based on the total population, we elucidated confounding of asthma medication on the observed results by repeating the main analyses after exclusion of 33 men reporting use of asthma medication for a period of a week or more during the three months prior to study participation.

Crude and adjusted β-coefficients with corresponding 95% confidence interval (CI) were estimated, with men without self-report of asthma serving as the reference. In analyses estimating the association between self-reported asthma and levels of reproductive hormones, the β-coefficients and 95% CIs were back-transformed to reflect the percentage difference in hormone concentration for men with asthma compared with the reference [19]. Due to the square-root and cubic-root transformation used for semen parameters, these results were illustrated as β-coefficients with a corresponding 95% CI on the transformed scale. *p*-values < 0.05 were considered statistically significant. All statistical analyses were performed in IBM SPSS 25.0 (IBM Corporation, Armonk, NY, USA).

## 3. Results

A total of 6177 men with a median age of 19 years were included in the study, of which 656 (10.6%) reported having ever been diagnosed with asthma. Overall, asthmatic and non-asthmatic men were largely comparable regarding anthropometric measures. However, the group of men reporting asthma more often reported that their mother had smoked during pregnancy, that they had used medication for at least one week during the three months prior to study participation, and that they had experienced later pubertal onset than non-asthmatic men (Table 1).

Generally, self-reported asthma appeared to be consistently associated with lower testicular function, assessed as semen quality and reproductive hormone levels, but with few substantial or statistically significant differences (Table 2 and Table 3). Compared with men with no self-report of asthma, men with self-reported asthma had similar semen volume (median: 3.1 vs. 3.2 mL), sperm motility (median: 61 vs. 62%), and morphology (median: 6.5 vs. 6.5%), but lower sperm concentration (median: 42 vs. 46 million/mL) and total sperm count (median: 133 vs. 145 million) (Table 2). These differences persisted in the adjusted regression analyses and were statistically significant for total sperm count (adjusted β: −0.18, 95% CI: −0.33 to −0.04 million on cubic root transformed scale, *p*-value: 0.01) and borderline statistically significant for sperm concentration (adjusted β: −0.09, 95% CI: −0.18 to 0.01 million/mL on cubic root transformed scale, *p*-value: 0.08) (Table 2). Men with and without self-reported asthma had similar reproductive hormone levels, with a general tendency to lower levels (except FSH) among men with self-reported asthma, based on the regression analyses, albeit with very small effect sizes (Table 3).

We detected no modifying effect of allergy on the association between self-reported asthma and total sperm count. Estimates of association were of similar magnitude among men with and without allergy (p_interaction_ = 1.0), not supporting any synergistic effects (Table 4). Excluding the 26 men with chronic bronchitis did not alter the results, nor did exclusion of the 33 men with current regular use of asthma medication.

## 4. Discussion

In this cross-sectional study of 6177 men, we found that men with self-reported asthma had lower sperm concentration and total sperm count compared with non-asthmatic men. The association between asthma and total sperm count was not modified by allergy, nor was the association explained by confounding by asthma medication use, since our sensitivity analysis excluding men with regular use of asthma medication showed that this did not change the observed association. However, the cross-sectional design of the study limits ascertainment of causality.

In a mouse model, asthma in male mice was related to reduced testicular function, including increased spermatocyte apoptosis, structural changes in spermatozoa, delay in sperm maturation, inhibition of Sertoli cell function, and reduced sperm count and motility [6]. However, the existing knowledge in humans is scarce. Our specific findings on the association between asthma and lower sperm concentration and total sperm count imply, for the first time, that an association between asthma and reduced testicular function in men may exist, which could potentially be caused by the inflammatory response [5]. Although no previous human studies have directly compared fertility or fertility markers in asthmatic and non-asthmatic men, studies have reported associations between other inflammatory diseases, such as rheumatoid arthritis, and male infertility or subfertility [20,21].

One previous study focusing on the potential adverse effect on testicular function of asthma medication, rather than asthma *per se*, reported that sub-fertile men using sympathomimetics (n = 32) had better semen quality, specifically sperm motility, than non-sympathomimetic-users (n = 850), which was retained with further adjustment for asthma to account for confounding by indication, suggesting that asthma did not explain the observed association [13]. However, the proportion of asthmatic men in the two groups is not evident from the paper, and the majority of sympathomimetic-users would be expected to have asthma, which is why adjustment for asthma may not be the most relevant approach. Therefore, interpreting the specific role of asthma in that previous study and directly comparing with results from our study is difficult. In contrast, our sensitivity analysis excluding men with regular use of asthma medication showed that this did not change the observed association between asthma and testicular function, which is why asthma medication is not thought to explain the reduced sperm count among men with ever-diagnosed asthma in our population, nor does it seem to improve it, as suggested by the previous study. However, due to the low number of men with regular use of asthma medication at the time of study participation (n = 33) more specific analyses of the impact of this type of medication could not be performed. Furthermore, sporadic use of medication was not included in our study and could still play a role.

We could not confirm any modifying effects of allergy on the association between asthma and testicular function. But we acknowledge that systemic inflammation in asthma is not fully understood, and that there are indications suggesting a relationship between systemic inflammation and elevated proinflammatory cytokines in the peripheral blood [22]. Cytokines are also relevant as paracrine regulators of spermatogenesis in the normal testis [23] and up-regulation of some specific cytokines during acute testicular inflammation can induce adverse effects on germ cells and disrupt spermatogenesis [23]. This is further supported by the fact that infection and inflammation of the male reproductive tract are important etiological factors of subfertility and infertility [23]. We only observed associations to sperm count, while no significant differences between men with and without asthma were observed for reproductive hormones levels. Reduced sperm count could be expected to also be reflected in lower Inhibin B and higher FSH levels, and the estimates also pointed in this direction for men ever diagnosed with asthma, but differences were minor. Inhibin B has previously been shown primarily to be a good spermatogenic marker in men with low Inhibin B levels but not across the entire range [24], which could explain the lack of association between asthma and Inhibin B in this population of young men from the general population. In general, semen quality is also associated with Leydig cell function, as men with lower semen quality also present with higher LH levels needed to sustain their testosterone level [25]. However, in this study, isolated associations to spermatogenic markers were observed without indications of an affected Leydig cell function, suggesting that the testes were not globally affected.

A key strength of this study is the large study population of men unselected regarding testicular function and considered representative of the background population with the assessment of semen quality and reproductive hormone levels as specific biomarkers of fertility potential. Furthermore, while critical disease outcomes based on register diagnoses are relevant, only severe cases of asthmatic disease and allergies will be diagnosed in a hospital setting, and thus, self-reported information about these diseases is considered more specific. This is supported by a Danish study comparing questionnaire data and data from the Danish National Patient Register, reporting an asthma prevalence of 12% and 7%, respectively [26].

Certain limitations warrant mentioning. First, the study was cross-sectional, hindering any conclusions on causation. Additionally, we do not have any biomarkers of asthmatic/systemic inflammation, and the hypothesized mechanism behind the observed association cannot be elucidated. Thus, the modest association observed between asthma and sperm counts may be explained by a common risk factor for both conditions rather than a direct effect of asthma on spermatogenesis. This could be a risk factor of prenatal origin, such as maternal smoking during pregnancy, predisposing for both asthma and impaired testicular function in the male offspring [14,27]. Or it could be a contemporary risk factor, such as air pollution, found to be associated with both conditions [28,29,30]. Next, the questionnaire did not include information on the severity or timing of asthma diagnosis, which is expected to affect the degree of inflammation, and the association between asthma and semen parameters may be stronger among men with current self-reported asthma compared with men with a prior diagnosis but potentially no current asthmatic symptoms. Due to the phrasing used in the questionnaire in the first part of the study period, men with chronic bronchitis were categorized as asthmatic cases. However, sub-analyses indicated that this had no impact on the results. Finally, there is a risk for chance findings, as multiple associations were investigated. Consequently, the findings in this study need validation. The study population was limited to young men, as the majority were between 18 and 23 years of age, and it would be relevant to assess whether the same association between asthma and testicular function is present in the age group where most men start family formation.

Our findings are of public health importance, as semen quality has been reported to be in decline over the past few decades [7,8], and asthma is prevalent in young men and may therefore be a contributing factor to the low sperm count. Therefore, further studies are needed to investigate the impact of asthma and asthma treatment, as well as the effect of optimizing treatment for asthma, to elucidate the association between asthma and semen parameters.

In conclusion, in this large cross-sectional study, self-reported asthma was found to be associated with reduced sperm concentration and total sperm count for the first time in humans. However, the cross-sectional design of the study limits ascertainment of causality. Future studies using a prospective cohort design with thorough assessment of severity of asthma, use of asthma medication, and assessment of inflammatory markers may further elucidate any potential association between asthma and testicular function.

## Figures and Tables

**Table 1:** Characteristics of the 6177 men cross-sectionally investigated in 2001–2020, presented as raw values [median (5–95 percentiles) or %], according to self-reported asthma status.

	No Asthma (n = 5521, 89.4%) Median (5–95 Percentile) or %	Asthma (n = 656, 10.6%) Median (5–95 Percentile) or %	*p*-Value ^c^
Anthropometric measures			
Age (years)	19.0 (18.4–22.0)	18.9 (18.4–22.8)	0.6
Height (cm)	182 (171–193)	181 (170–192)	0.08
Weight (kg)	73.5 (58.8–94.9)	73.5 (59.5–97.3)	0.5
BMI (kg/m^2^)	22.2 (18.4–28.4)	22.4 (18.7–28.7)	0.06
Mean testis size, OM (mL)	21 (14–30)	20 (13–29)	0.1
Mean testis size, US (mL)	14 (9–21)	14 (8-21)	0.1
Health and health behavior			
Ejaculation abstinence time (h)	62 (37–135)	62 (37–134)	0.7
Alcohol (mean units/week)	9 (0–37)	9 (0–35)	1.0
Cigarette smokers (yes),	45.5	47.1	0.4
Maternal smoking during pregnancy (yes)	32.8	37.7	0.01
Use of medication ^a^ (yes), %	12.4	21.0	<0.001
Voice change, %			0.2
Earlier than peers	19.3	18.1	
Later than peers	15.2	18.3	
Same time as peers	65.4	63.6	
Sexual dysfunction ^b^, %	8.4	8.3	0.3

OM = orchidometer, US = ultrasonography. ^a^ Use of any medication for a period of a week or more during the 3 months prior to study participation. ^b^ Answered “Often” to at least one of the questions regarding lack of interest in sex, problems with erection, premature ejaculation, or lack of ejaculation. ^c^ Differences between the two groups were calculated using Mann–Whitney *U* test for continuous variables and Chi^2^ test for categorical variables.

**Table 2:** Associations between self-reported asthma and semen parameters of 6146 men, cross-sectionally investigated 2001–2020, presented as raw values [median (5–95 percentiles)] and as results from linear regression analysis [β-coefficient (95% confidence interval, CI)].

			Crude		Adjusted ^d^	
	n ^c^	Median (5–95 Percentile)	β	95% CI	β	95% CI
Volume (mL) ^a^						
No asthma	5488	3.2 (1.4–6.1)	Reference		Reference	
Asthma	650	3.1 (1.3–5.9)	−0.03	−0.06; 0.01	−0.03	−0.06; 0.01
Concentration (million/mL) ^b^						
No asthma	5493	46 (4–158)	Reference		Reference	
Asthma	653	42 (3–158)	−0.09	−0.18; 0.01	−0.09	−0.18; 0.01
Total sperm count (million) ^b^						
No asthma	5487	145 (11–522)	Reference		Reference	
Asthma	650	133 (7–447)	−0.18	−0.33; −0.04	−0.18	−0.33; −0.04
Motile sperm (%) ^a^						
No asthma	5460	62 (26–80)	Reference		Reference	
Asthma	649	61 (22–80)	−0.03	−0.13; 0.08	−0.02	−0.13; 0.08
Morph. normal sperm (%) ^a^						
No asthma	5341	6.5 (1.0–16.0)	Reference		Reference	
Asthma	635	6.5 (0.5–15.5)	−0.04	−0.12; 0.03	−0.05	−0.12: 0.03

^a^ Square-root-transformed variable for linear regression. ^b^ Cubic-root-transformed variable for linear regression. ^c^ n does not add up to final study population on semen parameters (n = 6146) due to missing values for some semen parameters in some men. Furthermore, men with azoospermia are not included in motility or morphology analyses. ^d^ Adjusted for BMI, smoking, maternal smoking during pregnancy, ejaculation abstinence time. The model assessing the association between asthma and motility is additionally adjusted for duration between ejaculation to motility assessment.

**Table 3:** Associations between asthma and reproductive hormone levels of 6114 men, cross-sectionally investigated 2001–2020, presented as raw values [median (5–95 percentiles)] and as results from linear regression analysis [β-coefficient (95% confidence interval, CI)].

			Crude		Adjusted ^c^	
	n ^b^	Median (5–95 Percentile)	β (% Difference)	95% CI	β (% Difference)	95% CI
Follicle stimulating hormone (IU/L) ^a^						
No asthma	5458	2.6 (1.0–6.5)	Reference		Reference	
Asthma	648	2.6 (1.1–6.5)	2.42	−2.34; 7.41	2.61	−2.61; 7.61
Inhibin B (pg/mL) ^a^						
No asthma	5455	179 (87–314)	Reference		Reference	
Asthma	648	180 (87–308)	−2.75	−6.14; 0.80	−2.38	−5.77; 1.14
Luteinizing hormone (IU/L) ^a^						
No asthma	5464	3.4 (1.6–6.5)	Reference		Reference	
Asthma	650	3.3 (1.7–6.3)	−0.35	−3.79; 3.21	−0.70	−4.11; 2.94
Testosterone (nmol/L)^a^						
No asthma	5455	19.6 (11.6–32.3)	Reference		Reference	
Asthma	648	19.4 (11.4–33.2)	−0.92	−3.52; 1.75	−0.76	−3.33; 1.87
Free testosterone (pmol/L) ^a^						
No asthma	5451	448 (267–750)	Ref.		Ref.	
Asthma	648	443 (265–758)	−0.29	−2.97; 2.46	−0.51	−3.18; 2.22

^a^ Natural-logarithm-transformed variable for linear regression. β-estimates (95% CI) are back-transformed to express the percentage (%) difference. ^b^ n does not add up to final study population on hormonal parameters (n = 6114) due to missing values for some analyses in some men. ^c^ Adjusted for BMI, smoking, maternal smoking during pregnancy, time of blood sampling.

**Table 4:** Effect modification of self-reported allergies on the association between self-reported asthma and total sperm count of 3487 participants, cross-sectionally investigated 2008–2020.

Total Sperm Count (Million) ^a^					
Allergy	Asthma	N	β ^b^	95% CI	*p*-Value for Interaction
Yes	No	516	Ref.		1.0
	Yes	171	−0.27	−0.55; 0.02	
No	No	2598	Ref.		
	Yes	202	−0.27	−0.52; −0.02	

^a^ Cubic-root-transformed variable for linear regression. ^b^ Adjusted for BMI, smoking, maternal smoking during pregnancy, and ejaculation abstinence time.

## Data Availability

Data used in the present study is maintained centrally at Rigshospitalet and data access is regulated by national data protection rules. Thus, data are not publicly available and anonymized data can only be accessed after approval by relevant authorities.
